# Improving the Microstructural and Rheological Properties of Frozen Unfermented Wheat Dough with Laccase and Ferulic Acid

**DOI:** 10.3390/foods12142772

**Published:** 2023-07-21

**Authors:** Ni He, Zhiqin Pan, Lin Li, Xia Zhang, Yi Yuan, Yipeng Yang, Shuangyan Han, Bing Li

**Affiliations:** 1 School of Food Science and Engineering, Guangdong Province Key Laboratory for Green Processing of Natural Products and Product Safety, Engineering Research Center of Starch and Plant Protein Deep Processing, Ministry of Education, South China University of Technology, Guangzhou 510640, China; joyheni@163.com (N.H.); pan_scut@foxmail.com (Z.P.); lilin@dgut.edu.cn (L.L.); cexzhang@scut.edu.cn (X.Z.); yyuan@fzu.edu.cn (Y.Y.); yyp3460@163.com (Y.Y.); 2 School of Chemical Engineering and Energy Technology, Dongguan University of Technology, Dongguan 523808, China; 3 College of Biological Science and Engineering, Fuzhou University, Fuzhou 350108, China; 4 School of Biology and Biological Engineering, South China University of Technology, Guangzhou 510640, China

**Keywords:** laccase, ferulic acid, frozen unfermented dough, rheological properties, viscoelastic behaviors, protein conformation

## Abstract

The quality deterioration that is induced by freezing treatment limits the development of frozen dough technology for standardized and delayed baking. In this study, laccase (LAC) and ferulic acid (FA) were employed to improve the rheological properties and microstructure of frozen unfermented dough. The results showed that the dough with LAC + FA had a lower softening degree than the dough with FA alone. Correspondingly, LAC + FA incorporation enhanced the viscoelastic behavior of frozen unfermented dough with better stability. Furthermore, a more uniform and homogeneous gluten network was observed in the LAC + FA-supplemented dough after 21 d of storage. The structural stability of the frozen gluten sample increased after LAC + FA treatment, possibly owing to an increase in the oxidation degree of FA. Moreover, LAC + FA treatment promoted the oxidation of the sulfhydryl groups to some extent, resulting in more extensive cross-linking. LAC + FA treatment hindered the protein conformational changes typically induced by frozen storage compared with LAC alone. Overall, LAC + FA treatment has a synergistic effect on enhancing the viscoelastic behaviors of frozen unfermented dough and inhibiting the conformational variation in frozen gluten; thus, it shows promise for improving frozen dough.

## 1. Introduction

Frozen dough products have gained a growing interest in the market because of their standardization, convenience, and efficiency. At present, frozen unfermented dough is present in a large proportion of a variety of foods, especially Chinese traditional staple foods, such as noodles, dumplings, siu mai, and wontons. However, the destruction of the starch structure, the change in gluten network integrity, and the redistribution of moisture caused by freezing treatment affect the final quality of frozen dough products, limiting their further development potential [1]. In order to overcome these adverse effects induced by the freezing process, an effective and promising strategy is to add appropriate improving agents through which to strengthen the network structure [2].

The cross-linking of wheat flour gluten via inter- or intra-molecular covalent bonds is conducive to the formation of the three-dimensional network structure of the dough. Currently, transglutaminase [3], tyrosinase [4], and laccase (LAC) [5,6] are utilized to improve dough properties via promoting the cross-linking of gluten. Among them, LAC is a highly attractive enzyme because of its wide range of substrate specificity to polyphenols, methoxy-substituted phenols, and diamines [7]. Specifically, LAC is a copper-ion-containing polyphenol oxidase (EC 1.10.3.2) that can catalyze the oxidation of aromatic substrates and produce active radicals, which may cause cross-linkage between phenols and proteins [8]. Based on the LAC-mediated oxidation of dough constituents, Si et al. [9] proposed that LAC could be used as a dough- and bread-improving agent. Since then, the application of LAC in flour processing has attracted more and more attention. LAC was predominantly used to catalyze the oxidative cross-linking of feruloyl esters on the arabinoxylans (AX) of wheat dough but also promote the interaction between glutenin macropolymers and pentosan fractions [10,11]. In addition, Manhivi et al. [5] observed the altered viscoelasticity of taro dough with LAC and a concomitant decline in thiol and total phenols, which might indicate the formation of disulfide bonds and thiol–phenol conjugates in the dough. However, the complex spatial structure of the gluten hindered its accessibility to the active sites of LAC, thus preventing extensive cross-linking of the substrates [12,13]. Fortunately, this limitation can be overcome by adding a small molecule mediator capable of radical transfer reactions [12,13]. It has also been reported that reactive quinones are prone to interact with nucleophilic amino acid residues (methionine, lysine, tryptophan, and cysteine) within proteins [14]. Hence, there appears to be a great necessity for promoting cross-linking reactions by combining LAC with mediators (such as phenols), ultimately with beneficial effects on the rheological behavior of dough and the quality of the final product [15].

Ferulic acid (FA), a phenolic acid commonly found in plants, is one such mediator [16]. Labat et al. [17] found that exogenous feruloyl radicals induced by LAC could inhibit the cross-linking of AX through competitive binding with endogenous AX feruloyl esters and promote the formation of disulfides among proteins through the radical transference from phenoxy radicals to sulfhydryl groups. It has also been reported that the oxidation product of FA (e.g., semi-quinone), catalyzed by LAC, readily oxidized cysteine to cystine [18]. Furthermore, LAC could induce the cross-linking of tyrosine-containing peptides or proteins to form conjugates in the presence of FA [12,19]. It can be inferred that the synergistic effect of LAC combined with FA (LAC + FA) can hinder the damage from ice crystals by strengthening the cross-linking of gluten and the AX network, thus improving the properties of frozen unfermented dough.

The aim of this study was to investigate the effects of LAC and FA on the rheological properties and structural characteristics of frozen unfermented dough. For this purpose, farinographic, dynamic rheological, and tensile properties were utilized in order to assess the changes in the rheological properties of the dough. In addition, the variations in the spatial structure were investigated via scanning electron microscopy (SEM), Fourier transform infrared spectroscopy (FTIR), free sulfhydryl content, and intrinsic fluorescence spectroscopy. Attempts were made to further understand the protective effects of LAC and FA on frozen unfermented dough and facilitate their application in flour processing.

## 2. Materials and Methods

### 2.1. Materials

White wheat flour, containing 15.7% moisture, 12.3% protein, and 0.4% ash, was purchased from the New Land Group (Xinxiang, China). *T. versicolor* LAC was obtained from Sigma-Aldrich Chemicals (Shanghai, China). FA (≥99%) was purchased from Aladdin (Shanghai, China). Bradford protein assay kits were supplied by Nanjing Jiancheng Bioengineering Institute (Nanjing, China). Other chemicals used were of analytical or chemical reagent grade.

Preliminary work revealed that LAC (20 nkat/g flour) and FA (250 μg/g flour) resulted in doughs with optimal texture; thus, this formulation was used throughout. One nkat corresponds to the oxidation of 1 nmol of substrate per second.

### 2.2. Microfarinograph Measurement

LAC (20 nkat/g flour) and FA (250 μg/g flour), alone or in combination, were added to the flour (4 g, 14% moisture base) in order to investigate their effects on farinogram parameters, using a 4 g microfarinograph (Micro-Dough LAB, Perten Instrument, Stockholm, Sweden), including water absorption (percentage of water to yield the consistency of 500 Brabender Units (BU), development time (time to reach up to the consistency of 500 BU), stability time (duration that dough remained at a consistency of 500 BU), and softening degree (distance 12 min after the consistency of 500 BU was obtained). Each sample was carried out in duplicate.

### 2.3. Preparation of Dough Samples

Wheat flour (200 g), distilled water (120 mL), and/or LAC (20 nkat/g flour), and/or FA (250 μg/g flour) powder were mixed in the kneader (HM 750, Hauswirt, Qingdao, China) for 10 min until the surface of the dough was smooth, and the dough without LAC and FA was used as the control group. After resting for 5 min, the dough was divided into equal-weight pieces and wrapped with plastic film. The molded dough was quickly frozen at −80 °C for 10 min and stored at −18 °C for 0 or 21 d. Then, the samples were thawed at 30 °C and 70% relative humidity for 1 h. Some samples were used after freeze-drying, while others were directly used for subsequent experiments.

### 2.4. Rheological Measurement

The rheological characteristics of the dough were measured using a controlled-stress rheometer (Paar Physica MCR 302, Anton Paar, Graz, Austria) with parallel-plate geometry (25 mm diameter) according to the method described by Migliori et al. [20]. The thawed dough was placed in the center of a parallel plate with a gap of 1 mm, and the excess dough was removed. Vaseline was applied to the edges of the samples to prevent drying. After resting for 5 min, the oscillation test was performed at 25 °C, 1% strain (in the linear viscoelastic region), and a frequency range of 1–100 rad/s. The storage modulus (G′), loss modulus (G″), and loss tangent (tan *δ* = G″/G′) were recorded to reflect the rheological characteristics of the samples.

### 2.5. Kieffer Rig Uniaxial Extensibility

Dough extensibility was determined using a texture analyzer (TA-XT Plus, Stable Micro System, Surrey, UK) equipped with a Kieffer extensibility rig according to the method described by Kieffer [21]. The thawed dough was placed in a lubricated Teflon molder and compressed into dough strips. After resting for 20 min at 30 °C, a strip of dough was removed from the molder and clamped between the plates of the Kieffer rig prior to each test. The samples were tested in tension mode at 3.33 mm/s test speed and 75.0 mm distance.

### 2.6. Preparation of Gluten Solution

Gluten was prepared according to the Chinese National Standards GB/T 5506.1-2008 [22]. The dough was kneaded and washed with distilled water to remove the starch until an iodine solution failed to produce a blue color. The gluten powder was obtained from wet gluten by freeze-drying, grounding, and 60-mesh screening.

Frozen gluten samples were prepared as described by Wang et al. [23]. Gluten (44% *w*/*w*) was mixed with LAC, FA powder, or both using a kneader (HM 750, Hauswirt, China) for 10 min. All samples were then wrapped in a plastic film and left for complete hydration at 4 °C for 30 min. The freezing procedure was the same as that for frozen, unfermented dough. The obtained samples were freeze-dried for further analysis.

The gluten solution was prepared as described by Zhao et al. [24,25] with slight modifications. Freeze-dried gluten samples (400 mg) were placed in 50 mL of 500 mM acetic acid and continuously stirred for 24 h in an ice bath. Then, the extracted samples were centrifuged at 10,000 r/min and 4 °C for 30 min by a centrifuge (3-30KS, Sigma Laborzentrifugen GmbH, Osterode am Harz, Germany). According to the polymer theory, 500 mM acetic acid is a good dilution solution for gluten proteins since it does not influence the structure of the gluten molecular chain [24]. The protein concentration in the supernatant was measured using a Bradford protein assay kit.

### 2.7. Microstructure of Dough

The microstructure of the dough and gluten samples at different frozen storage times was observed using an SEM (EVO 18, Carl Zeiss AG, Mainz, Germany). The freeze-dried dough and gluten samples were sputter-coated with gold and observed under an acceleration voltage of 10 kV at a magnification of 1000 times.

### 2.8. Fourier Transform Infrared Spectroscopy (FTIR)

The FTIR spectra of the gluten samples were recorded using an FTIR spectrophotometer (Vertex 33, Bruker, Ettlingen, Germany) in the range of 400–4000 cm^−1^ at a resolution of 4 cm^−1^. According to the method of Yang et al. [26], a mixture of the dried gluten sample and anhydrous potassium bromide was thoroughly ground before being pressed into a transparent disc for FTIR analysis.

### 2.9. Measurement of Total Free Sulfhydryl Content

Ellman’s reagent was used to determine the free sulfhydryl content of gluten samples under different frozen-storage conditions, according to the method described by Liu et al. [27], with minor modifications. Briefly, 2 mL of 1 mg/mL gluten solution was mixed with 2 mL of Tris-glycine buffer containing 8 M urea (0.086 M Tris, 0.09 M glycine, 0.004 M EDTA, pH 8.0). Then, 200 μL of 4 mg/mL DTNB (5,5′-dithio-bis (2-nitrobenzoic acid)) was dissolved in the above buffer and added to the above mixture. After incubating at room temperature for 30 min, the absorbance of the solution was measured at 412 nm using a UV-VIS spectrophotometer (Carry 50, Varian, Palo Alto, CA, USA). The absorbance value was converted to the free sulfhydryl content using the standard curve of 0–0.2 mM cysteine.

### 2.10. Measurement of Intrinsic Fluorescence Spectra

The intrinsic fluorescence spectra of the protein samples were determined using a fluorescence spectrophotometer (F-7000, Hitachi, Tokyo, Japan) according to the procedure described by Wang et al. [28]. The gluten solution was diluted to 1 mg/mL using the extraction solvent. The excitation wavelength was 280 nm, and the emission wavelengths were recorded from 290 to 410 nm. Both the slit widths were set at 5 nm.

### 2.11. Statistical Analysis

All data were expressed as mean ± standard deviation (SD). The statistical significance level of *p* < 0.05 was derived via one-way analysis of variance (ANOVA) and Duncan’s test using SPSS 22.0 statistical software. The graphs were drawn with Origin 2021 software and GraphPad Prism 8.0 software. Unless otherwise stated, experiments were performed in triplicate, except for SEM and FTIR.

## 3. Results

### 3.1. Microfarinograph Analysis

Farinographs can be used to determine the mechanical resistance of dough during mixing and kneading, which is a crucial indicator of the processing performance of flour [29]. The farinogram parameters are shown in Table 1. Compared to the control samples, doughs with LAC or LAC+FA required more time to form the optimum dough, indicating a tough network. In addition, the stability time of the LAC-supplemented dough was longer than that of the control sample, suggesting a higher tolerance to mechanical shear (by the mixing blades) and enhanced gluten strength. In contrast, the FA treatment resulted in an increased degree of softening, revealing reduced resistance to mechanical stirring and weakened gluten strength. Notably, the addition of LAC + FA significantly inhibited the weakening effect of FA on the dough, suggesting that LAC could overcome the adverse effects of FA on the structural stability and improve the farinographic properties of the flour.

### 3.2. Dynamic Rheology Properties

**Figure 1:**
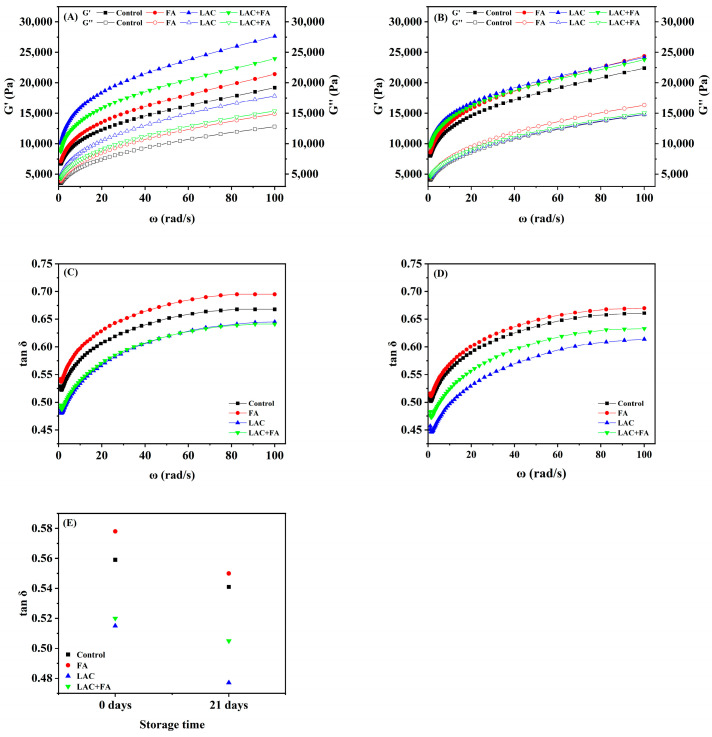
Effects of laccase (LAC) and ferulic acid (FA) on the rheological properties of frozen unfermented dough. (**A**,**C**) 0 d of frozen storage, (**B**,**D**) 21 d of frozen storage, (**E**) loss tangent (tan δ) at 1 Hz.

The three-dimensional network structure formed via the interaction between gluten and starch or non-starch polysaccharides endows the dough with viscoelasticity [30]. Thus, the frequency-dependent behavior of dough viscoelasticity was evaluated using small deformation oscillatory rheology, demonstrating the effect of LAC and FA on the interaction among the dough components [31]. Generally, G′ and G″ signify the elasticity and viscosity of the dough, respectively, whereas tan *δ* reflects the relative contribution of dough elasticity and viscosity [31]. As displayed in Figure 1, the dough behaves like a gel (G′ > G″). The differences in G′ and G″ were indicative of the short-range interactions in the dough, such as gluten–starch and starch–starch interactions. For all dough samples, as frozen storage time increased, G″ and G′ values decreased; G” values decreased more prominently in G′ values, resulting in an increase in tan δ. This suggested that the short-range interactions in the dough changed, leading to a decline of shear moduli upon shear deformations [32]. These results may be due to the structurally destructive water migration and ice crystal growth during the frozen storage period [33].

As shown in Figure 1E, when the frozen storage time increases to 21 d, the overall tan *δ* values of dough samples followed the order: LAC < LAC + FA < control < FA. In other words, LAC and LAC + FA contributed more to elasticity than to viscosity in frozen dough, whereas FA increased its viscosity. It could be inferred that LAC and LAC + FA altered the dough structure by enhancing the interaction among the dough components, thus delaying the deterioration of the frozen dough. Figueroa-Espinoza et al. [18] found that the semi-quinone of FA produced by LAC oxidation could oxidize cysteine and reduce glutathione while reducing itself to FA. Moreover, LAC could promote the cross-linking of AX through dimeric feruloyl esters [6]. This forms a strong AX network that acts on the gluten protein matrix, resulting in dough hardening [6].

### 3.3. Tensile Properties

**Figure 2:**
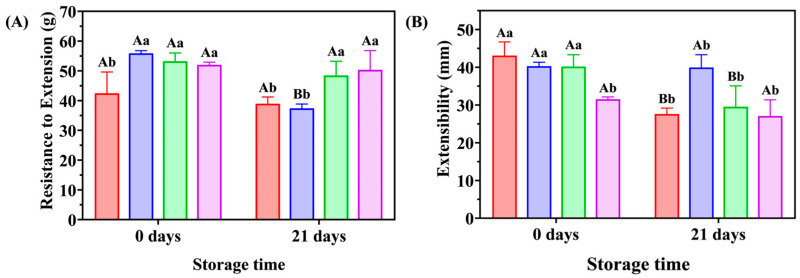
Effects of laccase (LAC) and ferulic acid (FA) on tensile properties of frozen unfermented dough. (**A**) Resistance to extension, (**B**) Extensibility (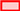, Control; 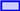, FA; 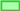, LAC; 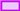, LAC + FA). The uppercase letters in the figure indicate significant differences between samples with the same ingredients, while the lowercase letters indicate significant differences within samples with the same frozen storage time (*p* < 0.05).

The individual and combined effects of LAC and FA on the tensile properties of dough were investigated by the Kieffer test [21]. In general, the maximum resistance to extension (R_max_) refers to the resistance of the dough when it is stretched to break, reflecting the dough’s strength [34]. The extensibility at maximum resistance (E_x_) refers to the stretch length of the dough when it breaks [35]. As shown in Figure 2A, compared with the control dough, a pronounced increase in R_max_ values is observed in the 0 d stored doughs with LAC, FA, or both; the increasing tendency persists in the R_max_ values of the 21 d-stored doughs with LAC and LAC + FA. As shown in Figure 2B, the E_x_ values of the control dough decreased with the extension of storage time, owing to changes in the dough structure caused by the freezing treatment [35]. In LAC-supplemented dough, the E_x_ value of the dough significantly decreased after 21 d of storage. Only LAC + FA induced a decline in the E_x_ value of 0 d-stored dough, which remained constant throughout the freezing period. It can be concluded from the above results that the structure of dough with LAC or FA was sensitive to freezing treatment, which might be due to the variation in the interaction among dough components under freezing conditions. However, LAC + FA treatment strengthened the tensile parameters of the dough and maintained its stability throughout the freezing period, suggesting that the synergistic effect of LAC and FA had a stabilizing impact on the viscoelastic behavior of frozen unfermented dough. These phenomena demonstrate that the combination of LAC and FA can enhance dough strength, thereby effectively reducing the deterioration of the dough structure caused by freezing.

### 3.4. Microstructure of Frozen Dough

**Figure 3:**
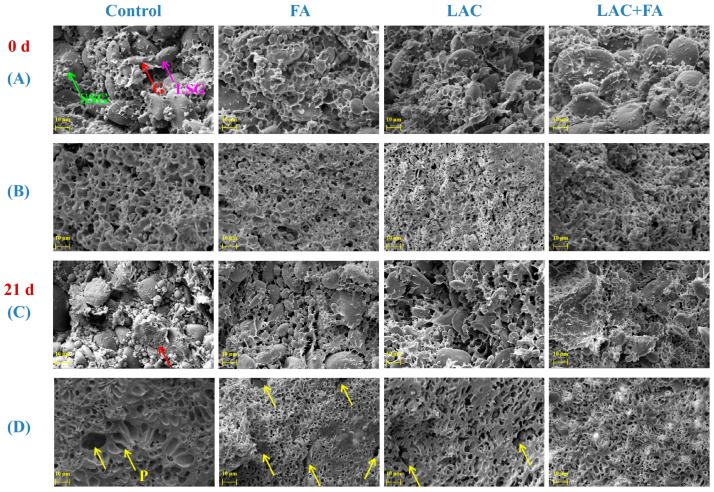
The scanning electron microscopy images of unfermented dough and gluten with laccase (LAC) and ferulic acid (FA) during frozen storage (×1000). (**A**,**C**) Unfermented dough samples, (**B**,**D**) Gluten samples. LSG (purple arrow): large starch granules, SSG (green arrow): small starch granules, G (red arrow): gluten network, and P (yellow arrow): pores.

The microstructural characterization of dough has contributed to expanding the effect of additives on its network, which plays a vital role in assessing the quality of the final products. The effects of LAC and FA on dough microstructure at different frozen storage times are shown in Figure 3. With prolonged storage, the gluten network of the control dough became thinner and more porous, which could explain the decrease in dynamic rheological properties and E_x_ values of the control dough mentioned above. The network of the 21 d stored FA-supplemented dough was relatively weak; irregular voids were observed in the gluten network, which might have allowed more starch granules to be separated from the gluten matrix. This corresponded to a sudden decrease in the R_max_ value of FA- supplemented dough after 21 d of storage (Figure 2A). Although LAC improved the gluten network of the 0 d-stored dough, disruptions persisted in the gluten matrix after 21 d of frozen storage. Additionally, the network structure of the 0 d-stored dough with LAC + FA was the most uniform and continuous, and the starch granules were tightly wrapped in a staggered gluten network, implying the enhanced integrity of the LAC + FA-supplemented-dough network. Interestingly, the gluten network of the frozen dough with LAC + FA remained homogeneous and compact after 21 d of storage. This suggested that FA, as a small molecular mediator, had a synergistic effect with LAC to promote the cross-linking of the gluten network.

### 3.5. Functional Groups Vibration Analysis

**Figure 4:**
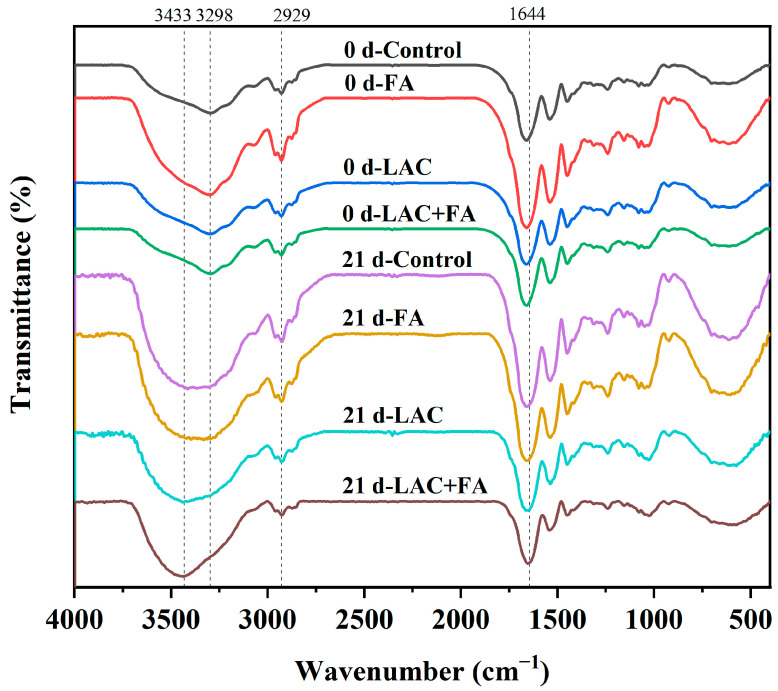
Effects of laccase (LAC) and ferulic acid (FA) on Fourier transform infrared spectroscopy of frozen gluten.

Changes in the functional groups and chemical bonds of gluten caused by LAC and FA during frozen storage were detected using FT-IR spectroscopy, as shown in Figure 4. The amide A band (3500–3000 cm^−1^) corresponds to N-H stretching coupled with hydrogen bonding, whereas the amide B band (3000–2500 cm^−1^) signifies C-H stretching and -NH_3_^+^ groups [36,37]. The addition of FA resulted in an enhanced signal at 3298 cm^−1^ (amide A band), indicating the formation of hydrogen bonds between the proton donor (FA) and proton acceptor (the carbonyl group of gluten). A similar phenomenon has been observed for proanthocyanidins bound to gliadins [38]. With prolonged storage time, the characteristic peaks of all samples shifted from 3298 cm^−1^ to 3433 cm^−1^, indicating that the freezing treatment altered the N-H stretching vibration. The peaks at 2929 cm^−1^ (amide B band) were related to C-H asymmetrical stretching [36]. In addition, the typical spectral properties of all gluten samples were in the amide region, including the amide I band (1700–1600 cm^−1^), denoting C-O stretching vibration or hydrogen bonds coupled with C-N stretching; amide Ⅱ band (1590–1500 cm^−1^), referring to N-H bending and C-N vibration; band (1490–1400 cm^−1^) indicating C-O stretching vibration; and amide Ⅲ band (1300–1000 cm^−1^), representing vibrations in the plane of C-N and N-H groups of bound amide or vibrations of CH_2_ groups of side-chains [36]. The ester-linkage band was located at 1644 cm^−1^, which could be overlapped with the amide I band [39]. The signal intensities of LAC and LAC + FA decreased at 1644 cm^−1^, possibly because of the higher degree of FA oxidation during AX gel formation [40]. Generally, there are two ways of FA dimerization: the self-crosslinking of AX feruloyl radicals [41] and cross-linking of FA radicals with nucleophilic residues in protein chains with subsequent FA covalent cross-linking [14]. It has been reported that dimer-FA and trimer-FA formed by LAC-mediated cross-linking of feruloyl esters lead to the formation of AX networks [42]. Moreover, reactive quinones catalyzed by enzymes are prone to interact with nucleophilic amino acid residues (methionine, lysine, tryptophan, and cysteine) in the protein chain, resulting in the formation of cross-linked proteins or polymers [14]. Among all samples, the signal intensity of gluten treated with LAC + FA did not change much during frozen storage, demonstrating that gluten treated with LAC + FA had higher structural stability, consistent with the SEM results.

### 3.6. Analysis of Free Sulfhydryl Content of Gluten

**Figure 5:**
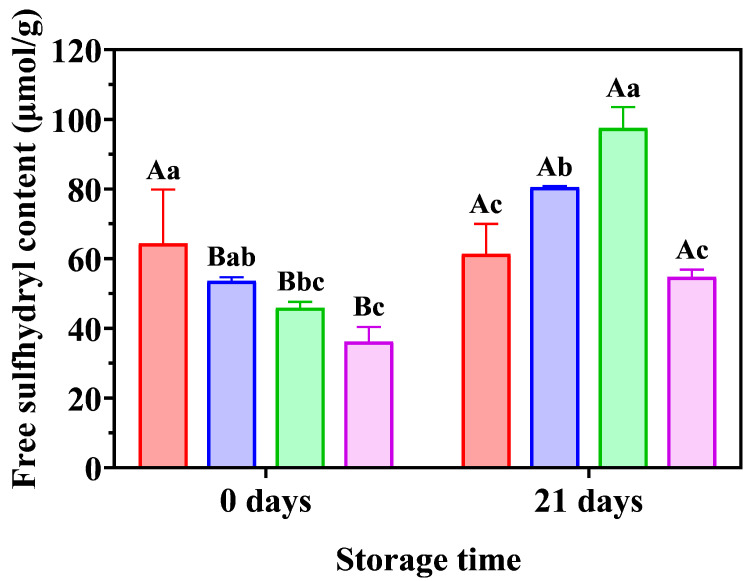
Effects of laccase (LAC) and ferulic acid (FA) on free sulfhydryl content of frozen unfermented dough (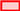, Control; 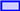, FA; 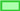, LAC; 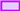, LAC + FA). The uppercase letters indicate significant differences between samples with the same ingredients, whereas the lowercase letters indicate significant differences within samples with the same frozen storage time (*p* < 0.05).

Glutenins are polymers linked by intra- or interchain disulfide bonds, whereas gliadins are a mixture of single chains with large intra-chain disulfide bonds. The formation or breakage of disulfide bonds is critical for the aggregation or depolymerization of gluten [24]. The effects of LAC and FA on the free sulfhydryl content of frozen gluten are shown in Figure 5. For 0 d storage, there was a slight decrease in the free sulfhydryl content of FA-supplemented gluten compared to that of the control gluten (*p* > 0.05), suggesting that FA might react with thiol radicals, resulting from disulfide bond breakage during mixing [43]. Moreover, LAC and LAC + FA reduced the free sulfhydryl content of 0 d-stored gluten compared to that of the control gluten. The LAC + FA treatment had the lowest free sulfhydryl content, indicating that the oxidation of sulfhydryl was accelerated by LAC in the presence of FA [17], which attributed to the synergistic effect of LAC and FA on the gluten. In addition, FA cannot directly oxidize cysteine, whereas its LAC-catalyzed oxidation product (semi-quinone) readily oxidizes cysteine to cystine [18]. This suggests that LAC-catalyzed FA (as a phenoxy radical) could further react with thiol compounds to form thiol–phenolic conjugates and disulfide bonds. A previous study found that LAC decreased the thiol and total phenol contents of taro dough, indicating that disulfide bonds and thiol-phenolic conjugates were possibly formed in the dough [5].

When the frozen-storage time was extended from 0 to 21 d, LAC, FA, and LAC + FA increased the free sulfhydryl content of the gluten samples to a certain level; however, no significant change in the free sulfhydryl content of the control gluten was observed. After storage for 21 d, the highest increase in the content of free sulfhydryl groups was observed in the dough containing LAC, which was probably due to the breakage of intra-disulfide bonds [44]. Previous studies have reported that FA-rich AX is present in gluten during fractionation [6] and that LAC could cross-link AX through dimeric feruloyl esters to form a strong AX network [6]. Wang et al. [44] found that although a higher glutenin macropolymer fraction was observed in the dough with water-extractable AX during the entire freezing period, its sulfhydryl content was more than that of the control dough due to the breakage of the intra-SS bonds. Thus, the quality of frozen steamed bread dough quality was enhanced. In the presence of FA, the effect of LAC on free sulfhydryl groups was diminished, suggesting that the synergistic effect of LAC and FA resulted in higher structural stability of gluten. In addition, FA increased the free sulfhydryl content of gluten compared to the other treatments, which is probably related to the reduced protein cross-linking and increased content of sodium dodecyl sulfate-soluble protein in the dough [45,46]. FA may affect the exchange reactions between disulfide bonds and free sulfhydryl groups, thus damaging the stability of the gluten network [47]. Interestingly, the synergistic effect of LAC and FA resulted in a lower increasing rate of free sulfhydryl content in frozen gluten compared with that in doughs with individual additions. Thus, enhanced viscoelasticity and stability of the frozen unfermented dough was achieved (Figure 2 and Figure 3). A reasonable hypothesis is that LAC promotes thiol oxidation in the presence of free FA. It is plausible that FA was oxidized by LAC to generate highly reactive phenoxy radicals in the thiol groups of proteins to form thiol radicals, thus forming disulfide bonds to facilitate gluten matrix cross-linking [18]. These phenomena are consistent with the results of the SEM analysis.

### 3.7. Analysis of Intrinsic Fluorescence Intensity

**Figure 6:**
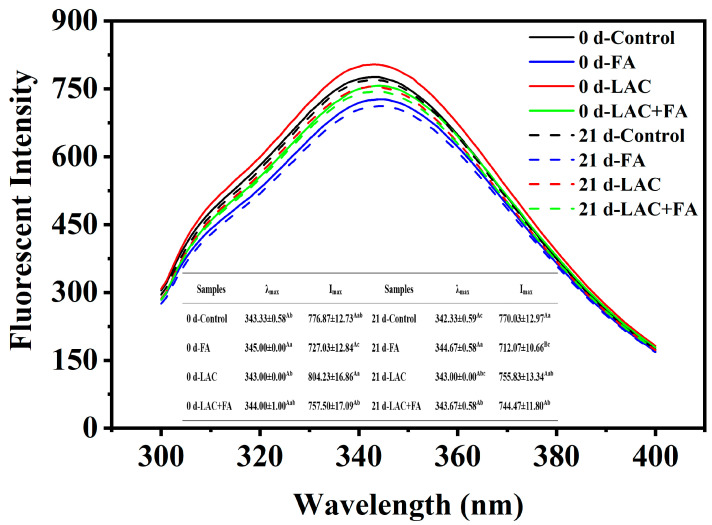
Effects of laccase (LAC) and ferulic acid (FA) on the intrinsic fluorescence intensity of frozen gluten. The uppercase letters indicate significant differences between groups, whereas the lowercase letters indicate significant differences within groups (*p* < 0.05).

Intrinsic fluorescence analysis was employed to characterize the intermolecular interactions and further confirm the conformational changes in gluten samples treated with LAC and FA alone or in combination. The fluorescence emission spectra of constituent chromophores, such as phenylalanine, tyrosine, and tryptophan residues, are particularly sensitive to their surrounding microenvironment [28]. As shown in Figure 6, FA caused the quenching of the intrinsic fluorescence of gluten samples accompanied by a red shift in the maximum fluorescence emission value (λ_max_) from 343 nm to 345 nm. This implied that the interaction of FA with gluten made the environment of tryptophan residues more hydrophilic. Similar fluorescence phenomena have been observed in FA interactions with rice protein or bovine serum albumin [48,49]. In contrast to the FA treatment, LAC treatment resulted in a notable increase in the fluorescence intensity of 0 d-stored gluten samples, which might be due to the partial masking of tryptophan residues by LAC-induced polymerization, aggregation, or peptide–peptide association [50]. The addition of LAC + FA had no significant effect on the fluorescence intensity of gluten throughout the frozen-storage period, which was attributable to the synergistic effect of LAC and FA. However, the decrease in the fluorescence intensity of gluten with LAC and LAC + FA subjected to freezing treatment indicated that the disturbed tertiary structure of the protein enhanced the environmental polarity of the fluorophores.

## 4. Conclusions

In this study, LAC and FA were used as compound modifiers to improve the rheological properties and microstructure of frozen unfermented dough. The results suggest that LAC + FA enhanced the viscoelasticity of frozen unfermented dough with better stability. Moreover, the gluten network of the frozen unfermented dough treated with LAC + FA was more continuous and uniform, and its conformational structure changed negligibly because of the increased oxidation degree of FA. In addition, LAC promoted thiol oxidation with the assistance of FA, possibly resulting in the formation of disulfide bonds and thiol-phenol conjugates which enhanced dough strength. Thus, LAC supplemented with FA has a synergistic effect on strengthening the viscoelasticity of dough and maintaining the stability of the gluten structure, which can ameliorate the adverse effects caused by frozen storage. Therefore, FA-assisted LAC is a promising agent for frozen dough improvement.

## Figures and Tables

**Table 1:** Effects of laccase (LAC) and ferulic acid (FA) on farinographic parameters of the dough.

Samples	Water Absorption (%)	Development Time (min)	Stability Time (min)	Softening Degree (FU)
Control	59.8 ± 1.1 ^a^	1.3 ± 0.1 ^b^	2.3 ± 0.1 ^b^	147.4 ± 3.5 ^c^
FA	58.8 ± 0.4 ^a^	1.3 ± 0.1 ^b^	2.0 ± 0.1 ^b^	204.9 ± 0.0 ^a^
LAC	60.5 ± 0.0 ^a^	1.9 ± 0.0 ^a^	3.7 ± 0.2 ^a^	132.5 ± 10.5 ^c^
LAC + FA	60.0 ± 2.1 ^a^	2.0 ± 0.3 ^a^	2.5 ± 0.4 ^b^	167.4 ± 3.5 ^b^

Data were expressed as the mean ± standard deviation (*n* = 2). Different superscript letters in same column indicate significance difference (*p* < 0.05).

## Data Availability

The data presented in this study are available upon request from the corresponding author.
